# Therapeutic Hemoglobin Levels after Gene Transfer in β-Thalassemia Mice and in Hematopoietic Cells of β-Thalassemia and Sickle Cells Disease Patients

**DOI:** 10.1371/journal.pone.0032345

**Published:** 2012-03-27

**Authors:** Laura Breda, Carla Casu, Sara Gardenghi, Nicoletta Bianchi, Luca Cartegni, Mohandas Narla, Karina Yazdanbakhsh, Marco Musso, Deepa Manwani, Jane Little, Lawrence B. Gardner, Dorothy A. Kleinert, Eugenia Prus, Eitan Fibach, Robert W. Grady, Patricia J. Giardina, Roberto Gambari, Stefano Rivella

**Affiliations:** 1 Department of Pediatrics, Division of Hematology-Oncology, Children's Blood and Cancer Foundation Laboratories, Weill Cornell Medical College, New York, New York, United States of America; 2 Dipartimento di Biochimica e Biologia Molecolare, Universita' di Ferrara, Ferrara, Italy; 3 Department of Molecular Pharmacology and Chemistry, Memorial Sloan Kettering Cancer Center, New York, New York, United States of America; 4 Red Cell Physiology Laboratory, New York Blood Center, New York, New York, United States of America; 5 Centro della Microcitemia e Anemie Congenite, Ospedali Galliera, Genova, Italy; 6 Department of Pediatrics, Albert Einstein College of Medicine, Children's Hospital at Montefiore, Bronx, New York, United States of America; 7 Department of Medicine, Montefiore Medical Center, Bronx, New York, United States of America; 8 Department of Pharmacology, Langone Medical Center, New York University, New York, New York, United States of America; 9 Department of Hematology, Hadassah–Hebrew University Medical Center, Jerusalem, Israel; 10 Department of Cell and Developmental Biology, Weill Cornell Medical College, New York, New York, United States of America; Emory University School of Medicine, United States of America

## Abstract

Preclinical and clinical studies demonstrate the feasibility of treating β-thalassemia and Sickle Cell Disease (SCD) by lentiviral-mediated transfer of the human β-globin gene. However, previous studies have not addressed whether the ability of lentiviral vectors to increase hemoglobin synthesis might vary in different patients.

We generated lentiviral vectors carrying the human β-globin gene with and without an ankyrin insulator and compared their ability to induce hemoglobin synthesis *in vitro* and in thalassemic mice. We found that insertion of an ankyrin insulator leads to higher, potentially therapeutic levels of human β-globin through a novel mechanism that links the rate of transcription of the transgenic β-globin mRNA during erythroid differentiation with polysomal binding and efficient translation, as reported here for the first time. We also established a preclinical assay to test the ability of this novel vector to synthesize adult hemoglobin in erythroid precursors and in CD34^+^ cells isolated from patients affected by β-thalassemia and SCD. Among the thalassemic patients, we identified a subset of specimens in which hemoglobin production can be achieved using fewer copies of the vector integrated than in others. In SCD specimens the treatment with AnkT9W ameliorates erythropoiesis by increasing adult hemoglobin (Hb A) and concurrently reducing the sickling tetramer (Hb S).

Our results suggest two major findings. First, we discovered that for the purpose of expressing the β-globin gene the ankyrin element is particularly suitable. Second, our analysis of a large group of specimens from β-thalassemic and SCD patients indicates that clinical trials could benefit from a simple test to predict the relationship between the number of vector copies integrated and the total amount of hemoglobin produced in the erythroid cells of prospective patients. This approach would provide vital information to select the best candidates for these clinical trials, before patients undergo myeloablation and bone marrow transplant.

## Introduction

β-thalassemia and SCD are two of the most common genetic red blood cell disorders, affecting millions. Although both conditions originate from genetic defects that reside within the β-globin gene, β-thalassemia is characterized by limited or absent synthesis of β-globin chains [1], whereas SCD by production of an aberrant β-globin molecule [2].

In β-thalassemia, mutant alleles that are associated with no β-globin synthesis are classified as β0, while those that allow some protein synthesis are designated β+. Thalassemic patients are therefore generally classified as β0/0, +/0 or +/+, based on the combination of these two alleles [1]. In SCD, the β chain is mutated at the sixth amino acid, leading to the synthesis of Hb S instead of normal Hb A [2]. The only definitive cure for both disorders requires transplantation of allogeneic bone-marrow (BM) cells, a procedure whose success depends on the availability of suitable donors and minimal development of graft versus host disease (GVHD) [3]. Therefore, therapies based on the modification of a patient's own BM cells by adding the corrected β-globin gene might offer a relatively safe alternative [3], [4], [5].

Several studies using thalassemic and SCD mouse models [5], [6], [7], [8], [9], [10], [11], [12], [13] support the use of lentiviral-mediated human β-globin gene transfer into autologous hematopoietic stem cells for the cure of these disorders [14], [15], [16]. Recently, the first clinical trial on a patient with Hb E/β-thalassemia was reported [17]. The success of this first trial was made possible by the additive effect of transgenic β-globin chains synthesized by the vector and those (fetal and adult) made by the patient's cells. Thus, without the support of endogenous hemoglobins (Hbs), the gene transfer would not have allowed this patient to become transfusion-independent. This result suggests that it would be extremely helpful if one could predict the outcome of gene transfer before a candidate undergoes myeloablation.

Here we describe a novel lentiviral vector, AnkT9W, that carries both the human β-globin gene and the erythroid-specific ankyrin 5′ hyper-sensitive (HS) barrier insulator [18]. This vector is able to maintain high, yet stable levels of Hb synthesis in MEL cells and in thalassemic mice. Furthermore, using just 30 mL of blood, we developed a protocol for evaluating the correlation between the number of AnkT9W-viral integrants (vector copy number, VCN), and that of β-globin mRNA molecules and the level of Hb production in peripheral-blood-derived human CD34^+^ and erythroid progenitor cells (ErPCs), following *in vitro* β-globin gene transfer and erythroid differentiation.

## Results

### The ankyrin insulator increases hemoglobin synthesis in murine erythroleukemia (MEL) cells

**Figure 1:**
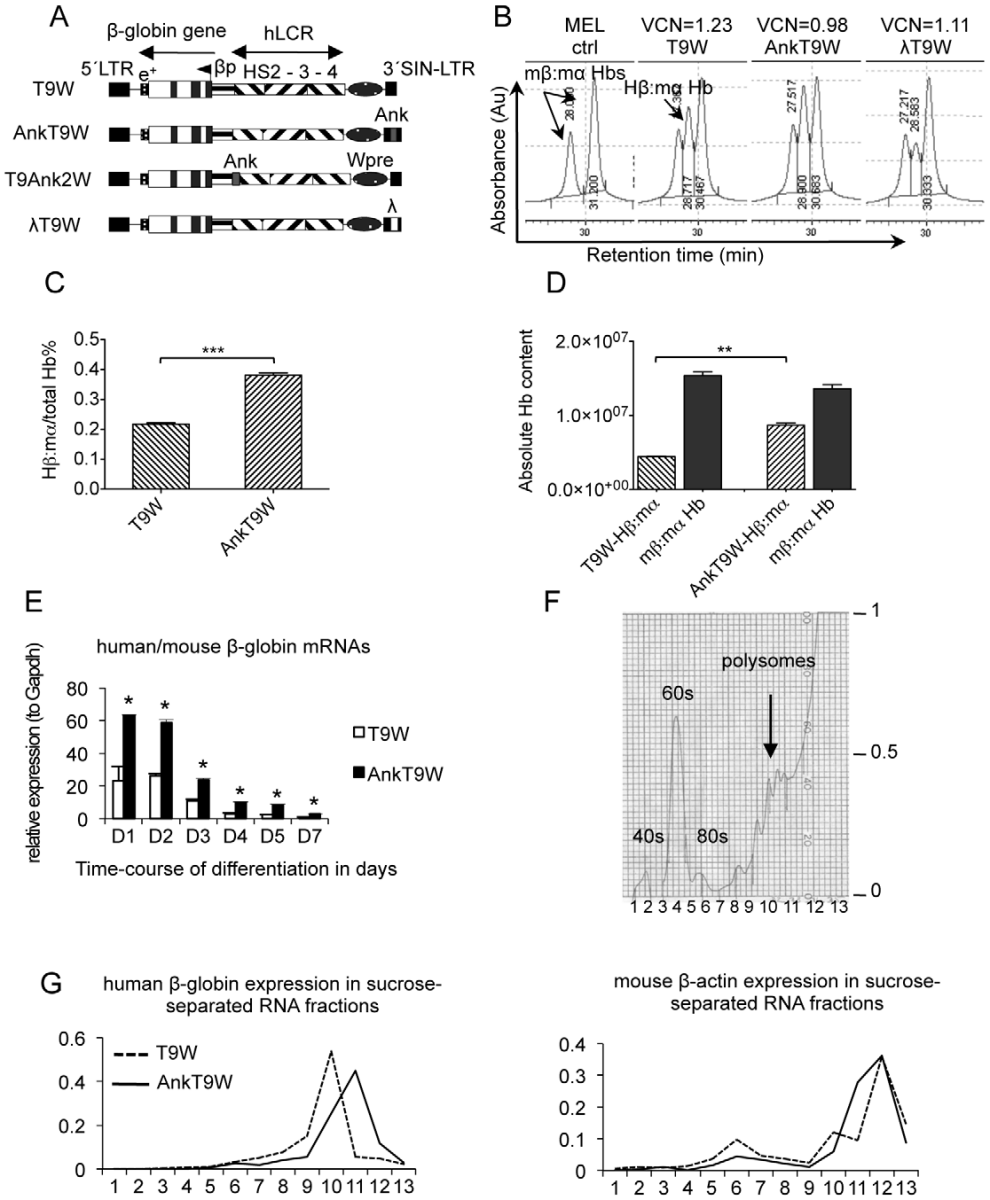
Lentiviral constructs and Hβ:mα Hb synthesis in MEL cells after lentiviral vectors integration. (A) Black and white rectangles represent exons and introns, respectively, within the β-globin gene. The human ankyrin insulator (in grey, 190 bp) was introduced into the 3′LTR, thereby generating AnkT9W. In the T9Ank2W vector we inserted the ankyrin element between the HS2 and the β-globin promoter. As a control for AnkT9W, the λT9W vector was generated, by replacing the ankyrin element with a fragment from the bacteriophage λ (190 bp, in white). βp: β-globin promoter. E^+^: β-globin enhancer. LCR: locus control region. HS: DNase I hypersensitive sites. LTR: long terminal repeat. SIN: self-inactivating. (B) HPLC analyses of murine Hbs (tetramers of two α-chains associated with two β minor/major chains) in control MEL cells, and chimeric human/mouse Hb (Hβ:mα) in MEL cells infected with approximately one copy of T9W (Hβ:mα = 30.7%), AnkT9W (Hβ:mα = 36.2%) and λT9W (Hβ:mα = 19.16%), respectively. The percentages and absolute amounts of Hβ:mα Hb after HMBA differentiation, in pools of MEL cells carrying three copies of T9W or AnkT9W are shown in C and D, respectively. (E) Expression of human/mouse β-globins expressed over a seven-day differentiation period in MEL cells carrying 3 copies of either T9W or AnkT9W. Values of both globins are normalized by mouse *Gapdh* expression. (F) Representative polysomal profile obtained. Continuous OD_254_ of 10–50% sucrose gradient with peaks corresponding to the 40 S, 60 S, 80 S and polysomal complexes are indicated. (G) Analyses of ribosomal RNA expression. Total mRNA of MEL cells carrying approximately 3 copies of T9W or AnkT9W was separated into 13 fractions after sucrose gradient ultra-centrifugation. Polysome-bound RNAs were collected between fractions 10 and 13 and represent messenger RNA with higher rate of translation. Values of mouse β-actin or human β-globin expression of each fraction were calculated as proportion of the total RNA.

A lentiviral vector named TNS9 carrying the human β-globin gene and large elements of the locus control region (LCR) dramatically reduced anemia in thalassemic mice [6], [9], [10]. As previously reported [19], we modified the vector backbone to increase its safety and efficiency, thereby generating T9W (Figure 1A).

Insulator elements are known for their (i) enhancer blocking activity, when placed between an enhancing element and a promoter [20], [21], [22], [23], and (ii) chromatin barrier activity (i.e, preventing the spread of heterochromatin into the integrated transgenic cassette from a nearby heterochromanized chromosomal region). The 5′ HS ankyrin insulator exhibits only the latter activity [18], which could prevent susceptibility to transcriptional silencing [24] and, in turn, increase overall expression. In order to evaluate whether the ankyrin insulator increases the probability of transgenic β-globin expression at random chromosomal integrations, we modified T9W by including a copy of this element [18] (190 bp) in the 3′ SIN-LTR, generating AnkT9W. As a control we generated a vector in which a fragment of DNA (∼190 bp) from the λ bacteriophage replaced the ankyrin element (λT9W). We analyzed pools of MEL cells transduced with T9W, AnkT9W and λT9W that exhibited comparable VCNs shortly after transduction. The percentages of chimeric human/mouse Hb over total Hb (Hβ:mα/(mβ:mα+Hβ:mα)) detected by HPLC was 30.7%, 36.2% and 19.2% in pools of MEL cells infected with T9W, AnkT9W and λT9W, respectively (Figure 1B). In two pools of MEL cells with similar copies of T9W and AnkT9W, both the percentage (Figure 1C) and absolute amount of Hβ:mα (Figure 1D) were higher in those cells carrying the AnkT9W vector. In order to investigate the cause of increased β–globin synthesis in AnkT9W transduced cells, we further characterized two pools of MEL cells carrying three copies of either T9W or AnkT9W, respectively (Figures 1E–G). We measured the relative amounts of transgenic human versus endogenous mouse β-globin mRNA expressed over the course of erythroid differentiation induced by HMBA (N,N′-hexamethylene-bis acetamide). Quantitative PCR analysis revealed that AnkT9W expressed the transgenic mRNA at a higher rate than T9W, especially during the early phases of erythroid differentiation (Figure 1E). In a clinical trial, monitoring the presence of the integrated vector and the expression of the transgenic mRNA over time is extremely important for interpreting the outcome of the gene transfer. For this reason, we modified the β-globin coding sequence with silent mutations that do not alter the amino acid residues but allow for discrimination between endogenous and transgenic β-globin DNA and RNA. We modified several bases in the first and second exons of separate vectors, generating AnkT9Wsil1 and AnkT9Wsil2 (Table 1 shows the modified sequences for each vector within exon 1 and 2). As a first step to compare AnkT9W, T9Wsil1 and AnkT9Wsil2, we repeated the measurement of the relative amounts of transgenic human versus endogenous mouse β-globin mRNA during erythroid differentiation in MEL cells, and observed that both these vectors increased expression of the transgenic β-globin gene during erythroid differentiation, as already seen with AnkT9W (not shown).

**Table 1:** Sequences of exon 1 and 2 in AnkT9W and AnkT9Wsil1/2 silent mutants.

**Canonic exon 1 (inside AnkT9W):** ATGGTGCACCTGACTCCTGAGGAGAAGTCTGCCGTTACTGCCCTGTGGGGCAAGGTGAACGTGGATGAAGT**T**GG**T**GG**T**GAGGCCCTGGGCAG
**Silent mutant exon 1 (inside AnkT9Wsil1):** ATGGTGCACCTGACTCCTGAGGAGAAGTCTGCCGTTACTGCCCTGTGGGGCAAGGTGAACGTGGATGAAGT**C**GG**C**GG**C**GAGGCCCTGGGCAG
**Canonic exon 2 (inside AnkT9W):** GCTGCTGGTGGTCTACCCTTGGACCCAGAGGTTCTTTGAGTCCTTTGGGGATCTGTCCACTCCTGATGCTGTTATGGGCAACCCTAAGGTGAAGGCTCATGGCAAGAAAGTGCTCGGTGCCTT**TAGT**GATGGCCTGGCTCACCTGGACAACCTCAAGGGCACCTTTGCCACACTGAGTGAGCTGCACTGTGACAAGCTGCACGTGGATCCTGAGAACTTCAGG
**Silent mutant exon 2 (inside AnkT9Wsil2):** GCTGCTGGTGGTCTACCCTTGGACCCAGAGGTTCTTTGAGTCCTTTGGGGATCTGTCCACTCCTGATGCTGTTATGGGCAACCCTAAGGTGAAGGCTCATGGCAAGAAAGTGCTCGGTGCCTT**CTCC**GATGGCCTGGCTCACCTGGACAACCTCAAGGGCACCTTTGCCACACTGAGTGAGCTGCACTGTGACAAGCTGCACGTGGATCCTGAGAACTTCAGG

Original and corresponding mutated bases of exons 1 and 2 inside constructs are in bold letters.

Increased β-globin protein with AnkT9W could be a result of increased translation and/or increased transcription. Polysomal analysis (Figures 1F–G) indicated that β-globin mRNA transcribed from AnkT9W co-localized with the polysomal fraction associated with the highest translational activity (Figure 1G, left). As expected, there was no difference between the translation efficiency of endogenous globin (data not shown) and β-actin mRNAs expressed by either AnkT9W or T9W transduced cells (Figure 1G, right). Together, these results suggested that the presence of the ankyrin insulator, irrespective of its localization in T9W, leads to increased β-globin mRNA and translation throughout erythropoiesis. These observations indicate that early expression of the transgenic β-globin messenger may be crucial in maximizing gene-therapy potential and efficacy in thalassemic erythroid cells.

### The ankyrin element is stably integrated and not subject to rearrangement in MEL cells carrying a single copy of the human β-globin transgene

**Figure 2:**
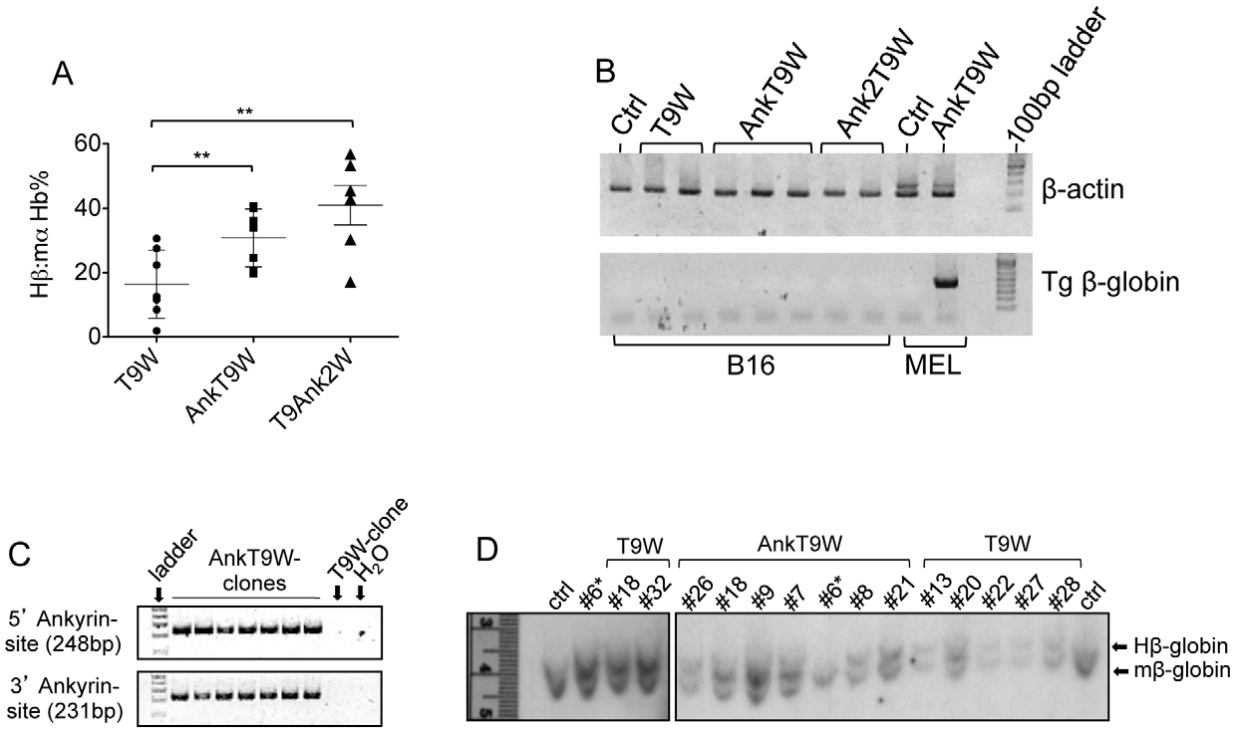
The ankyrin insulator improves translation of the β-globin gene, and its sequence is conserved at the site of integration. (A) Expression of the chimeric Hb (expressed as a percentage) after HMBA differentiation, in MEL cell clones carrying one VCN of T9W or AnkT9W, and T9Ank2W. One way anova test, p = 0.048. (B) The β-globin mRNA (bottom panel), expressed by T9W (VCN = 0.87,1.4), AnkT9W (VCN = 0.39, 1.07, 1.47) and T9Ank2W (VCN = 1.26, 1.98) was not detected in non-erythroid cells (B-16 melanoma) but only in differentiated MEL cells (AnkT9W, VCN = 2), indicating that the transgene's transcription is tissue specific. (C) Two amplicons were amplified by PCR corresponding to the ankyrin sequence present, respectively, in the 5′ and 3′ viral LTRs. Oligonucleotides were designed to include the ankyrin element and selectively amplify fragments either from the 5′ or 3′ LTR. Only clones bearing a single AnkT9W integrant were used. The amplified fragments exhibited the expected size of 248 bp and 231 bp, indicating that the ankyrin element was faithfully replicated in both LTRs. (D) Southern blot assay of genomic DNA from single integrant clones of MEL cells carrying either T9W or AnkT9W or from MEL control. The genomic DNAs were digested with *Xmn*I restriction enzyme, which yielded the full β-globin cassette, identified by hybridization using a β-globin *BamH*I-*Nco*I probe. Clone #6 of the AnkT9W series was reloaded in higher quantity (left panel) to amplify the signal of the transgenic human β-globin gene, because it was insufficiently loaded and exhibited a weak signal on the first blot (right panel).

We generated 14 clones of MEL cells carrying single copies of T9W (N = 7) and AnkT9W (N = 7), and analyzed their Hb content at 6 weeks or more and after HMBA-induced differentiation. In addition, to investigate whether the ankyrin insulator had any enhancer-blocking activity with respect to β-globin gene expression, we generated single copy MEL clones (N = 6) using a vector, T9Ank2W, in which this element was inserted between the β-globin promoter and the LCR (Figure 1A). Four out of 7 clones bearing T9W synthesized low amounts of human β-globin protein. In contrast, all the AnkT9W-bearing clones expressed the β-globin transgene at high levels. On average, the AnkT9W-bearing clones synthesized 46.6% more Hβ:mα (Figure 2A) than the T9W-bearing ones. The clones bearing the T9Ank2W exhibited increased β-globin expression, similar to those generated with AnkT9W. This indicates that the ankyrin element bears no enhancer blocking activity, but still exerts a positive effect on β-globin gene expression when located in proximity to the β-globin promoter and LCR. Tissue-specific expression of the β-globin transgene was assessed in pro-erythroid (MEL) and non-erythroid (B-16) cell lines (Figure 2B). Other vectors including insulator elements have been plagued by low titers or recombination events. In our hands the ankyrin element did not decrease the viral titer (comparing T9W vs. AnkT9W). We then investigated if the integration of the ankyrin insulator in 3′ LTR was associated with recombination events in the two LTRs as well as in the cassette including the β-globin gene and transcriptional elements. To this end, we verified that each ankyrin insulator sequence was stably integrated in both the 3′ and 5′ LTR of 7 MEL clones bearing a single copy of AnkT9W. Using specific primers that discriminate between the ankyrin sequence in the 3′ or 5′ LTR, we observed that each clone amplified two sequences of the expected size (Figure 2C). In addition, Southern blot analysis indicated that all the clones carrying one copy of either T9W or AnkT9W exhibited a 6.2 kb fragment, including the β-globin gene and all the transcriptional elements (Figure 2D).

### The vector carrying the ankyrin element improves correction of the phenotype of thalassemic mice

**Figure 3:**
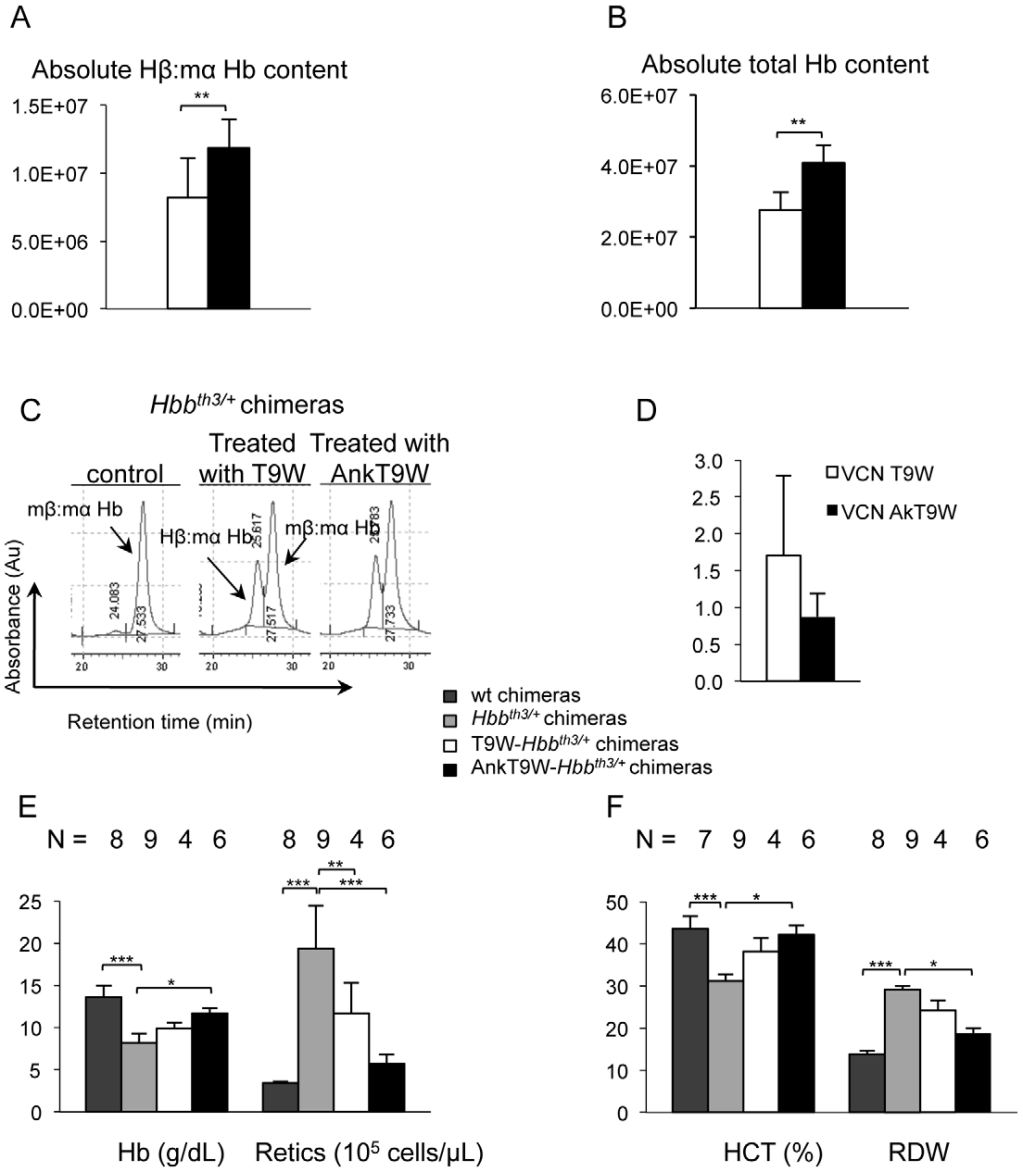
AnkT9W improves Hb synthesis and hematological parameters of thalassemic mice. Red cell hemolysates were analyzed monthly by HPLC for up to 6 months post BM transplant. Chimeric (A) and total absolute Hb content (B) in T9W-*Hbb^th3/+^* and AnkT9W-*Hbb^th3/+^* chimeras (n = 4 and 6, respectively). Hβ:mα Hb detected by HPLC and relative VCN (C and D) in mice chimeras obtained by transplanting *Hbb^th3+^* BM treated with T9W or AnkT9W vectors into lethally irradiated *Hbb^th3+^* recipient mice. (E, F) Hematologic parameters in WT, *Hbb^th3/+^*, T9W-*Hbb^th3/+^* or AnkT9W-*Hbb^th3/+^* chimeras. On average, the hematocrits in AnkT9W-treated chimeras were 26% and 10% higher, the reticulocyte counts 71% and 49% lower, and the RDWs 35% and 22% lower than in *Hbb^th3/+^* and T9W-*Hbb^th3/+^* mice, respectively. For reticulocyte counts, Hb, RDW, p<0.0001,and for HCT, p = 0.0002.

To compare the ability of the T9W and AnkT9W vectors to produce Hb *in vivo*, we engrafted mice affected by thalassemia intermedia (*Hbb^th3/+^*) [25], [26] with *Hbb^th3^* ^/+^ BM cells transduced with either T9W or AnkT9W. Figures 3A, B show the absolute Hβ:mα and the total Hb (Hβ:mα together with endogenous mouse Hb) content reached at 3 months and later in *Hbb^th3^* ^/+^ mice engrafted with either T9W- or AnkT9W-treated *Hbb^th3^* ^/+^ BM cells, respectively.

**Figure 4:**
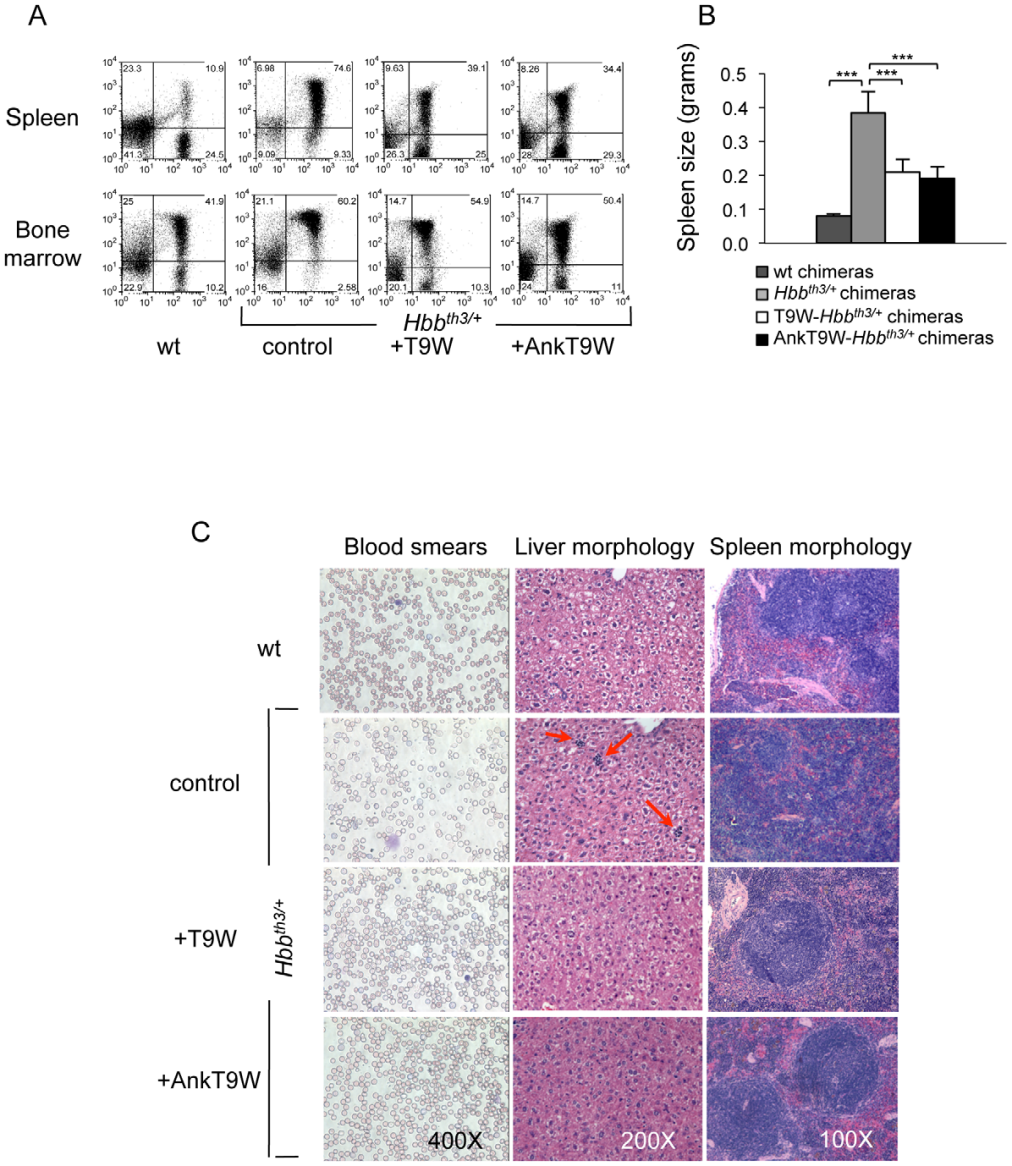
Hematopoietic parameters, splenomegaly and EMH of AnkT9-treated *Hbb^th3/+^*- are comparable to those of WT-chimeras. (A) Flow cytometry. CD71/Ter119 antibodies (y/x axes, respectively) were utilized to assess the correction of ineffective erythropoiesis in representative WT and *Hbb^th3/+^* mice and in *Hbb^th3/+^* mice treated with T9W or AnkT9W lentiviral vectors, in spleen (top) and bone marrow (bottom). (B) Mice spleen size. (C) From top to bottom: a WT mouse transplanted with WT bone marrow; an *Hbb^th3/+^* that received *Hbb^th3/+^* bone marrow untreated or transduced with T9W or AnkT9W. Liver and spleen morphology of the same animals are shown in the middle and right panels, respectively. (C, left panel) Blood smears stained with May-Grunwald-Giemsa staining, showing the RBCs morphology of representative mice. Sections of liver and spleen were also analyzed to assess extramedullary hematopoiesis (EMH) and organ morphology. The livers from both T9W- and AnkT9W-treated chimeras were similar to those of normal control mice in which no EMH was detected (C, middle panel). In the spleens of *Hbb^th3/+^* mice, the cross-sectional area of the white pulp (C, right panel) was relatively decreased and the marginal zones were obscured by the large number of nucleated RBCs, reflecting major expansion of the red pulp and erythroid precursors. In mice treated with T9W, and even more so in AnkT9W-treated chimeras, the amount of red pulp was decreased considerably, accounting for only about 40% to 50% of the cross-sectional area, and denoting a considerable reduction of EMH.

Compared to mice transplanted with un-manipulated *Hbb^th3/+^* BM, the chimeric and total hemoglobin contents were increased and red cell counts were significantly improved in all chimeras carrying AnkT9W. Compared to *Hbb^th3/+^* controls, the increases in average Hb values were 1.7 and 3.4 g/dL for T9W-*Hbb^th3/+^* and AnkT9W-*Hbb^th3/+^* mice, respectively. The ability of AnkT9W to increase Hb synthesis was supported by the observation that these parameters were corrected at relative low VCNs (0.9±0.3 and 1.7±1.1 in the AnkT9W-*Hbb^th3/+^* and T9W-*Hbb^th3/+^* chimeras, respectively; Figures 3C, D). The hematocrit, reticulocyte count and red cell distribution width (RDW) in AnkT9W-*Hbb^th3/+^* chimeras were nearly identical to those of mice engrafted with normal bone marrow (Figures 3E, F). Both T9W-*Hbb^th3/+^* and AnkT9W-*Hbb^th3/+^* chimeras exhibited a lower percentage and absolute number of immature erythroid cells (CD71^+^/Ter119^+^) than mature ones (CD71^−^/Ter119^+^, Figure 4A) and significantly reduced splenomegaly (Figure 4B), compared to *Hbb^th3/+^* untreated chimeras. Compared to *Hbb^th3/+^* and T9W-*Hbb^th3/+^* chimeras, AnkT9W-*Hbb^th3/+^* chimeras showed a more remarkable correction of their RBC morphology (microcytosis, hypochromia and poikilocytosis; Figure 4C, left panel), while organ morphology in the liver (Figure 4C, middle panel) and spleen (Figure 4C, right panel) were similarly corrected.

### Long-term expression of chimeric hemoglobin is sustained in secondary and tertiary chimeras transplanted with AnkT9W-treated *Hbb^th3/+^* bone marrow

**Figure 5:**
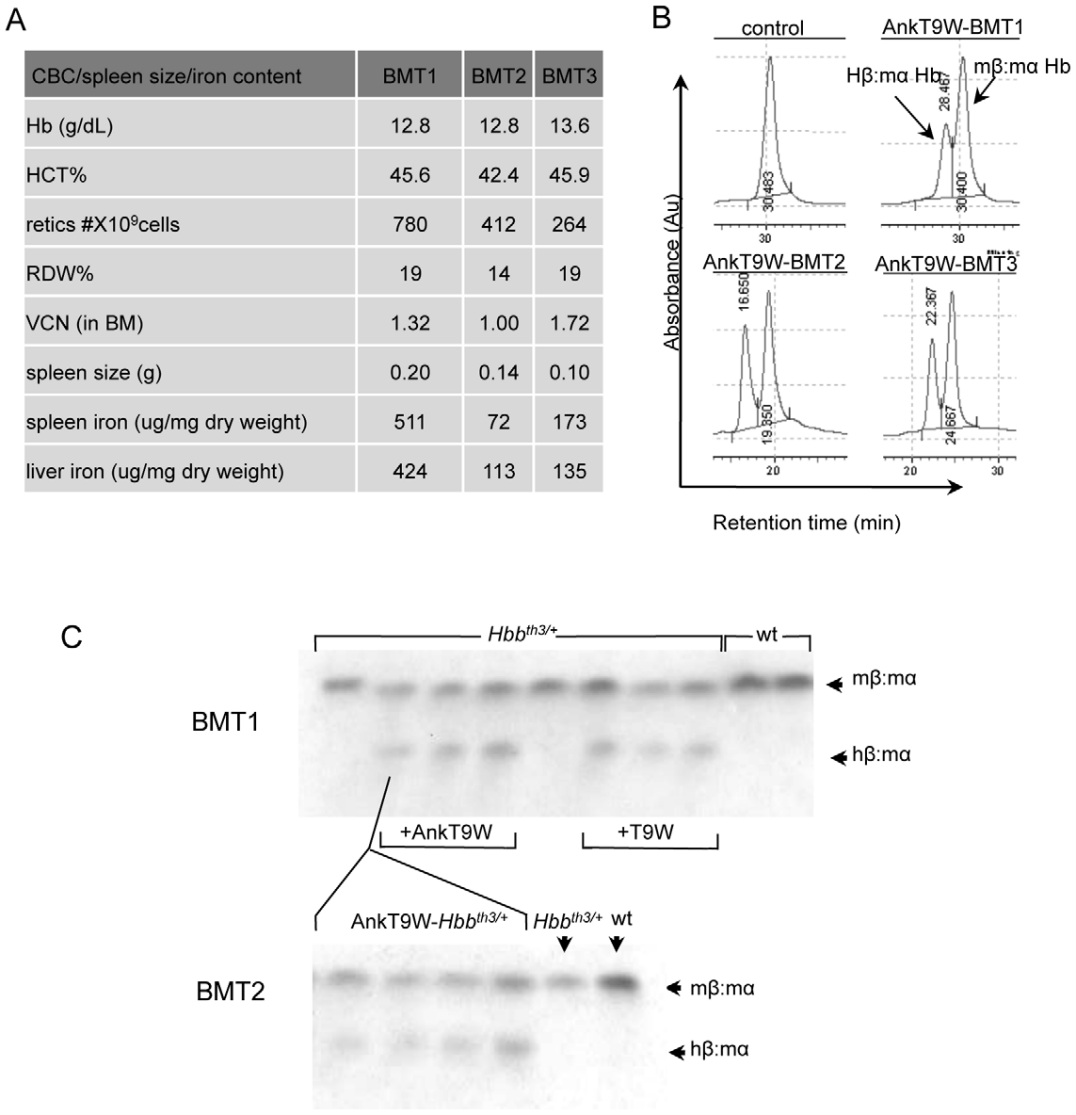
Long-term expression of Hβ:mα Hb and correction of the phenotype in thalassemic mice. (A) Hematological parameters, spleen size, and iron content in primary, secondary and tertiary chimeras. (B) HPLC profiles of *Hbb^th3+^* control mouse (upper left quadrant) compared to primary (right upper quadrant), secondary (left lower quadrant) and tertiary (right lower quadrant) BMT chimeras transplanted with AnkT9W-treated BM cells. (C) Cellulose acetate of primary (top) and secondary (bottom) BMT chimeras.

To assess long-term synthesis of β-globin in thalassemic hematopoietic cells transduced with AnkT9W, we collected the bone marrow of a primary chimera and transplanted it into an *Hbb^th3/+^* recipient and then repeated the process so as to establish a tertiary chimera. In the latter mice, we measured the level of chimeric hemoglobin (using both HPLC and cellulose acetate electrophoresis essays), hematological indices, the iron content of the liver and spleen (Figures 5A–C), and the proportion of immature/mature erythroid cells in the spleen (data not shown). The expression of chimeric hemoglobin in both secondary and tertiary chimeras was comparable to that of the primary chimera as were all other parameters, confirming that a sustained correction of the phenotype can be achieved utilizing AnkT9W.

### Amelioration of the phenotype in thalassemic human ErPCs treated with AnkT9W

Since AnkT9W showed an increased ability to express the transgenic β-globin gene in MEL cells and a more efficient long-term correction of the thalassemic phenotype in mice, we chose AnkT9W to study the correlation between viral integration and phenotypic correction of human thalassemic cells. In patients with β-thalassemia, the amount of β-globin synthesis is the major determinant of phenotype. ErPCs were isolated from peripheral blood and then expanded and differentiated via a modified version of the two-phase liquid culture system [27]. Compared to controls, the percentages of Hb A (α_2_: β_2_ tetramer) were 53% in β+/+ cells (N = 8), 17% in β+/0 cells (N = 6), and almost undetectable in β0/0 cells (N = 5) (not shown). Moreover, we eluted and characterized (via mass spectrometry) a peak observed in the HPLC profiles of thalassemic patients and positioned between those of the Hb Fs and Hb A. This analysis indicated that this peak corresponded to α-globin aggregates. This peak was not detected in any of the healthy control or carrier samples. The average amounts of α-globin aggregates were 9±5% in β+/+ cells, 8±5% in β0/+ cells, and 23±14% in β0/0 cells. The corresponding amounts of Hb F varied from 22±11% in β+/+ cells, to 54±23% in β0/+ cells, and 53±18% in β0/0 cells. The list of all patients analyzed in this study and their mutations is reported in Tables 2 and 3.

**Table 2:** Mutations in patients.

Group	Patient ID	Mutations
β0/0 (A)	1A	Nt168/Nt168
	2A	Codon6/Nt168
	3A, 5A	IVS-I-1/IVS-I-1
	4A	IVS-II-1/Nt 168
β0/+ (B)	1B, 2B, 3B	IVS-I-110/Nt168
	4B	IVS-I-5/Codon 15
	5B	IVS-II-654/Codon71–72
	6B	IVS-I-I/IVS-II-745
β+/+ (C)	1C	IVS-I-6/IVSI-110
	2C, 3C, 5C	IVS-I-110/IVSI-110
	4C, 6C	IVS-I-6/IVSII-745
	7C	IVS-I-6/Nt-30
	8C	IVS-II-745/IVS-II-745

List of β-thalassemia specimens analyzed in this study.

**Table 3:** Mutations phenotype.

β Type	Mutation ID	Category of defect
β0	Nt168/β039 (C→T)	Nonsense Mutation producing nonfunctional mRNA
	Codon 71–72 (+A)	Frame-shift Mutation producing nonfunctional RNA
	IVS-I-1 (G→A)	Splice site junction mutation
	IVS-II-1 (G→A)	Splice site junction mutation
	Codon 15 (TGG→TGA)	Nonsense Mutation producing nonfunctional mRNA
β+	IVS-I-5 (G→T)	Processing mutant-Mutation at the splicing consensus site
	IVS-I-6 (T→C)	Processing mutant-Mutation at the splicing consensus site
	IVS-I-110 (G→A)	Mutations Creating Alternative Splice Sites
	IVS-II-654 (C→T)	Mutations Creating Alternative Splice Sites
	IVS-II-745 (C→G)	Mutations Creating Alternative Splice Sites
	−30 (T→A)	Promoter Mutations-ATA Box

Description of β0 and β+ mutations.

**Figure 6:**
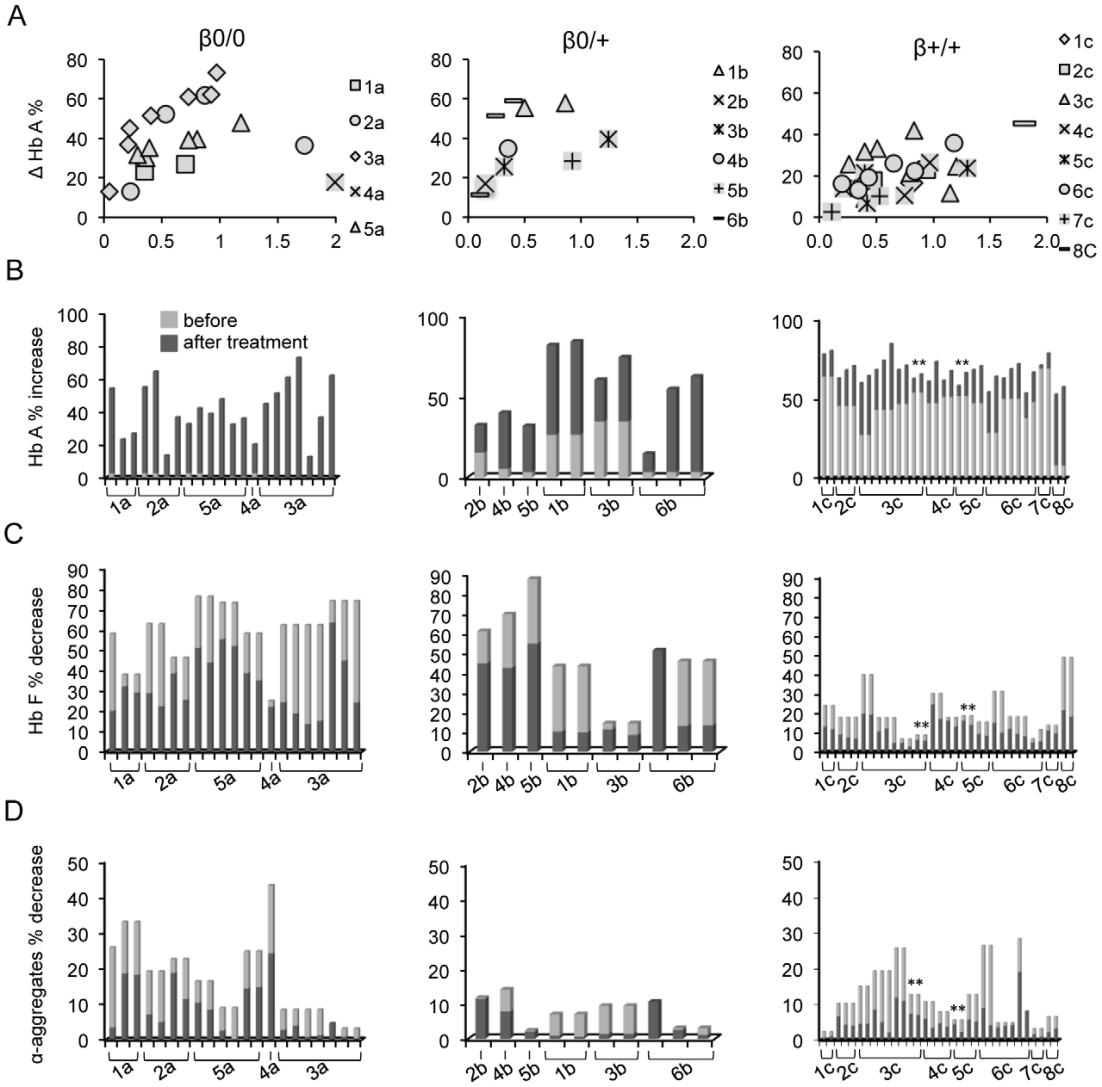
AnkT9W increases HbA synthesis to therapeutic levels in most of the thalassemic ErPCs. (A) Net increase of Hb A % (ΔHb A, calculated by subtracting Hb A after treatment from that following treatment) is plotted against VCN (on the X axis) in the three patients groups, β0/0, β0/+ and β+/+. The total Hb A% is also represented as percentage of Hb A before (light grey) and after transduction (dark grey) with AnkT9W in the same groups (B). Figures C and D show the concomitant percentages of Hb F and α-aggregates, both of which are reduced in thalassemic ErPCs after treatment with AnkT9W. The tie bars under the X axes group different specimens from the same individual. Several specimens were harvested and transduced at different times so as to expose the variability in such experiments. “*” Sign indicates specimens treated with AnkT9Wsil2.

**Figure 7:**
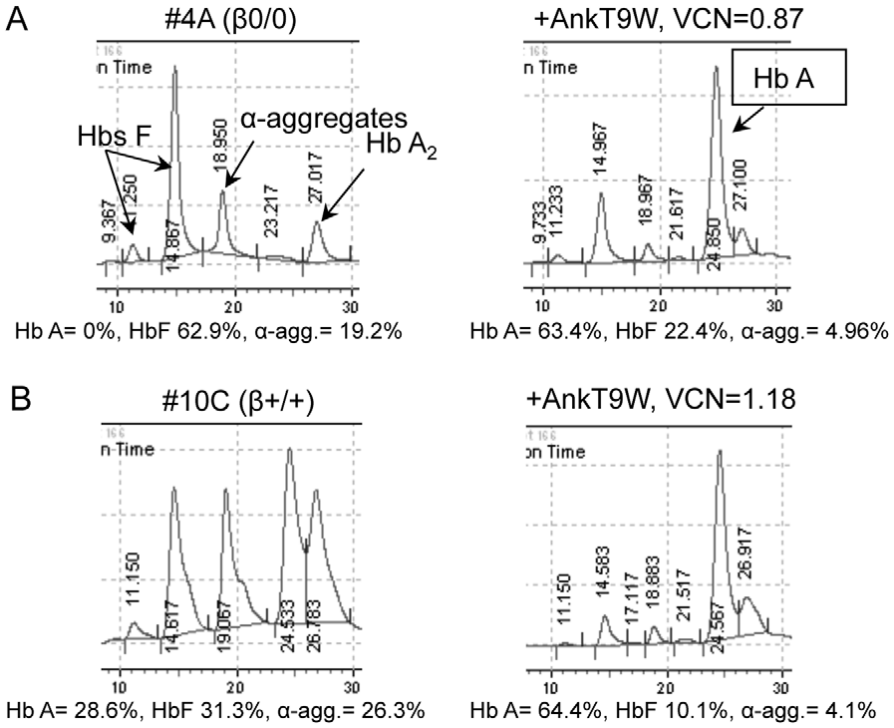
HPLC profiles of thalassemic specimens analyzed after differentiation from ErPCs-derived cultures. HPLC profiles from β0/0 (A) and β+/+ thalassemic ErPCs (B) at steady state (left), and after treatment (right) with AnkT9W. F, A, A_2_ (α_2_δ_2_) Hbs and α-aggregates are indicated by arrows.

We then determined the optimal conditions for infecting the cells with AnkT9W so as to achieve a VCN between 0.1 and 2, which is the expected range of transduction in a clinical setting. Using the same approach we also tested the ability of AnkT9Wsil2 to increase the levels of adult hemoglobin in a small subset of patient ErPCs, showing that the silent mutation did not alter the ability of the transgenic mRNA to generate adult hemoglobin (Figure 6B, ErPCs samples transduced by AnkT9Wsil2 are indicated by the “*” sign). The variation of Hb A content after treatment versus the number of integrations is shown in Figure 6A. Following transduction of ErPCs from β0/0 patients (on left, N = 5), we observed an increase in Hb A proportional to the VCN (ranging from 0.1 to approximately 1.0; with more than 95% of the cells hemoglobinized as indicated by benzidine staining). In Figure 6B the total percentage of Hb A is shown before and after treatment. Concomitantly, the percentages of fetal hemoglobins (Figure 6C) and α-globin aggregates (Figure 6D) were dramatically reduced. Representative HPLC profiles of untreated (left) and AnkT9W-treated (right) β0/0 and β+/+ samples are shown in Figures 7A and B, respectively.

**Figure 8:**
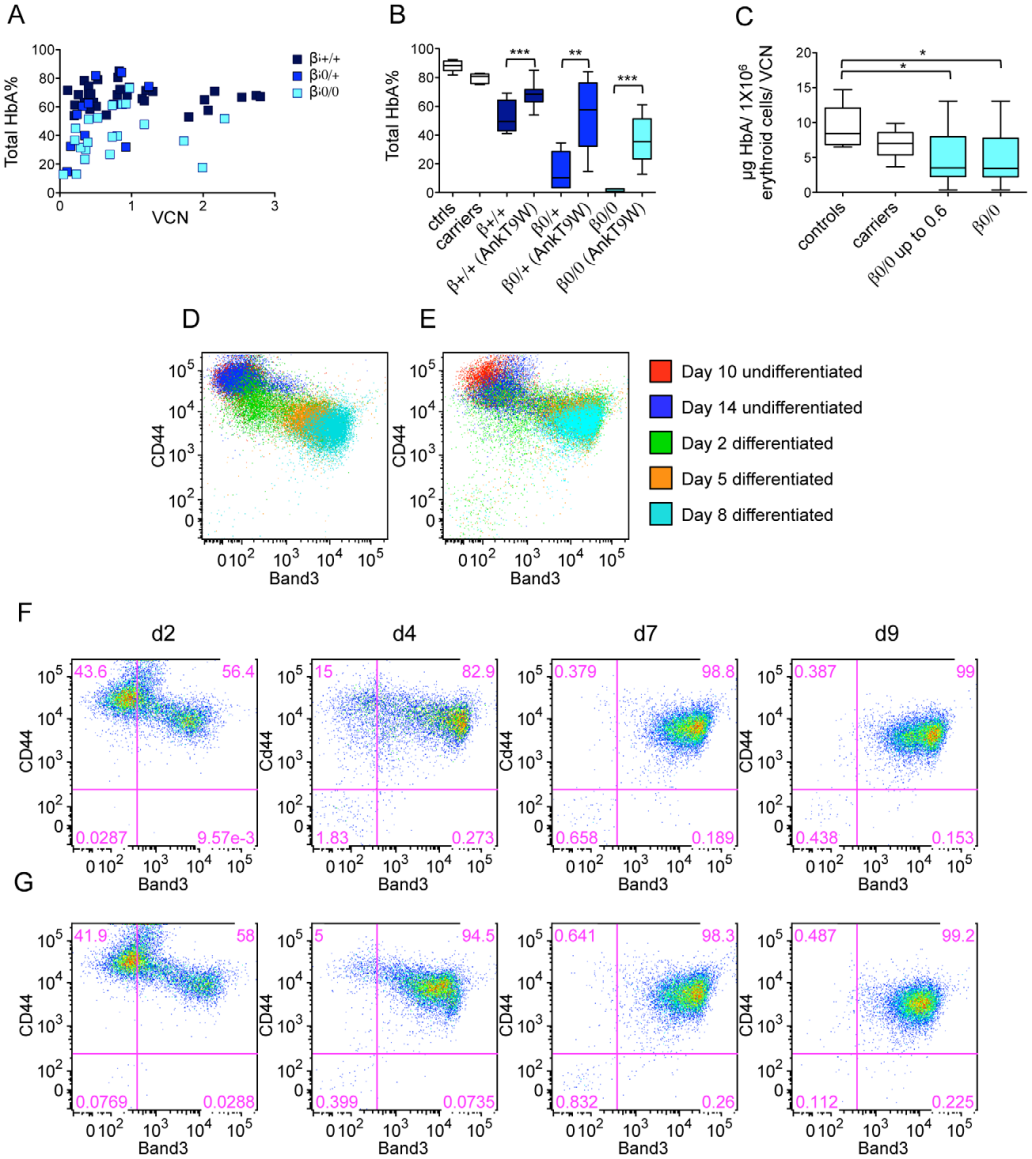
AnkT9W increases Hb A synthesis in thalassemic specimens derived from CD34^+^ cells, maintaining erythropoietic development unaltered. Figure A shows the percentage of total Hb A versus the number of AnkT9W integrations in the three major specimen groups. In figure B the Hb A% in ErPCs of thalassemic patients measured at baseline and after treatment with AnkT9W (VCN up to 0.6 copies) is compared to those of carriers and controls. In figure C the absolute Hb A content of 1×10^6^ erythroid cells in the carrier, control and β0/0 groups (normalized to the VCN) is indicated. Flow cytometry analysis of Band3/CD44 expression in normal (D) or thalassemic (E) CD34 cells from steady state and throughout erythroid differentiation. The expression of the two markers was analyzed on day 2-4-7 and 9 in β0/0 thalassemic cells untreated (F) or transduced with 0.92 copies of AnkT9W (G). The profiles did not differ throughout differentiation in the two experimental conditions, although HbA synthesis improved dramatically (see Figure 9).

Interestingly, although the VCNs in transduced β0/+ (N = 6) and β+/+ cells (N = 8) were similar to those in β0/0 cells, the net percentage increases of Hb A were less dramatic. In fact, up to a VCN equal to one, linear regression analysis revealed that β0/0 cells exhibited a higher response relative to the number of integrations (r squared = 0.47, p = 0.017), than β0/+ cells (r squared = 0.11, p = 0.35) or β+/+ cells (r squared = 0.12, p = 0.047). On the other hand, the total Hb A% in both β0/+ and β+/+ cells, which takes into account both endogenous and lentiviral-induced Hb, was higher than in β0/0 cells after transduction as shown in Figure 8A. This suggests that it might be possible to reach therapeutic levels of Hb A% in both β+/+ and β0/+ cells at low VCN. Furthermore, we observed that the total Hb A% reached a plateau in many patient specimens at relatively low VCNs. For these reasons, we narrowed down our analysis to samples exhibiting VCNs equal to or less than 0.6. Using this arbitrary threshold, the total amount of Hb A synthesized in β0/+ and β+/+ cells after transduction was comparable (54%±23% and 68%±7%, respectively) to that measured in carrier cells (80%±3%) and significantly higher (p<0.0001) than that produced by β0/0 cells (36%±16%) (Figure 8B). On the other hand, our analysis indicates that AnkT9W reaches complete therapeutic levels only in β0/0 specimens exhibiting the highest VCNs. Altogether, these observations suggest that β0/+ and β+/+ patients are more likely to achieve therapeutic levels of Hb A than β0/0 patients at integration levels that reduce genotoxicity risk and myeloablation regimens in a clinical setting. We also measured the absolute Hb A content at baseline and after treatment in the three groups of thalassemic cells, as well as in healthy controls and carrier cells. After treatment with AnkT9W, the average absolute amount of HbA in 1×10^6^ erythroid cells was increased in all groups, rising from 0 to 2.97±1.91 µg in β0/0, from 0.24±0.16 µg to 2.37±2.29 µg in β0/+ and from 2.72±1.47 µg to 5.03±1.92 µg in β+/+ cells. In order to correlate the absolute increase of Hb A after treatment with the VCN, we focused on β0/0 cells, which do not express any endogenous Hb A. The amount of Hb A produced by AnkT9W-treated β0/0 cells (normalized to 1 VCN) corresponded to 76% of that synthesized by carrier ErPCs (Figure 8C). This suggests that in this group one copy of AnkT9W might not be sufficient to raise Hb A to the levels necessary to achieve transfusion independence.

### CD34^+^ cells isolated from the peripheral blood of thalassemic patients express high levels of Hb A after treatment with AnkT9W

**Figure 9:**
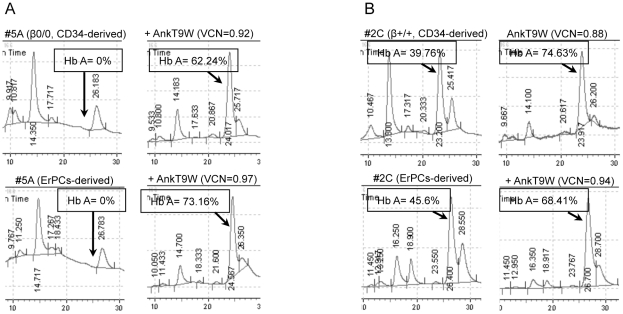
HPLC profiles of thalassemic specimens analyzed after differentiation from CD34-derived cultures. The HPLC profiles of the same thalassemic β0/0 (A) and β+/+ (B) specimens were analyzed at steady state (left) and following treatment with AnkT9W (right) after differentiation starting from either CD34^+^ cells (top) or ErPCs (bottom). In the same β0/0 specimen, AnkT9W contributes to increasing Hb A synthesis from from 0% to 62% (VCN = 0.92), in the CD34^+^-derived cells or to 73% (VCN = 0.97), in the ErPCs-derived cells. In the cells from the β+/+ patient, the net Hb increase was 35% (VCN = 0.88), if CD34^+^-derived or 23% (VCN = 0.94), if ErPCs-derived.

In order to maximize our pre-clinical approach, we developed a method to expand, differentiate and treat CD34^+^ cells *in vitro* following isolation from the blood via selective immunomagnetic separation. We used a modified version of the expansion system described by Leberbauer and colleagues [28]. This method has the advantage of generating a massive number of undifferentiated CD34^+^ cells that can be variously manipulated (i.e. frozen, thawed, infected, and analyzed for viral integration). Erythroid development was monitored using CD44 [29], Band3 and glycophorin A (CD235a) surface markers (Figures 8 D–G). Our results show that undifferentiated cells express high levels of the CD44 marker and are Band3^−^/CD235a^−^. As differentiation progressed, both control and thalassemic cells gradually became Band3^+^ and CD235a^+^, while the mean fluorescence of the CD44 marker regressed, as previously observed by Chen and colleagues [29] in the erythroid maturation of BM-derived murine CD34^+^ cells. The profiles of untreated and treated thalassemic cells did not differ throughout differentiation, although Hb A synthesis improved dramatically (from 0% to 62%, with a VCN = 0.92, as shown in Figure 9A and B).

### CD34^+^ cells derived from SCD patients and treated with AnkT9W Increased expression of Hb A to therapeutic levels

Gene therapy using lentiviral vectors encoding a normal β-globin gene may also provide a definitive cure for patients affected by SCD. However, one limitation of this approach is due to the fact that the β-globin encoded by the vector must replace the mutant β-globin chain in the adult hemoglobin tetramer made by the patient's red cells, without increasing the total amount of β-globin protein produced. We next investigated whether AnkT9W could increase the synthesis of Hb A without increasing the total amount of Hbs synthesized in SCD cells.

**Figure 10:**
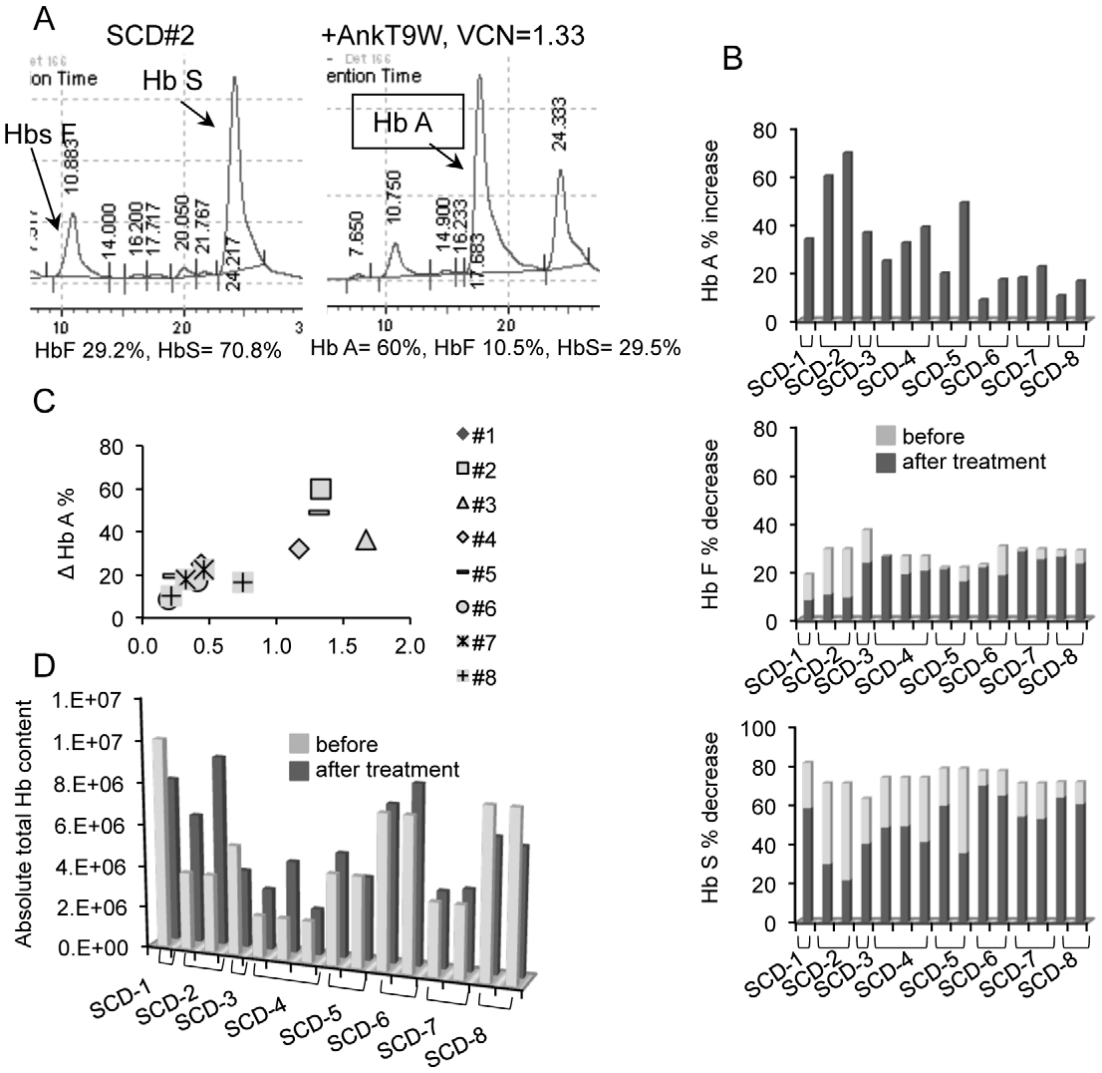
AnkT9W increases Hb A synthesis in SCD specimens derived from CD34^+^ cells, maintaining total hemoglobin content unaltered. (A) HPLC profile of a SCD specimen at steady state (left) or after treatment with AnkT9W (right). (B) Hb A% increase (top) and concomitant Hb F% (middle) and Hb S% (bottom) decrease after treatment with AnkT9W. (C) Increase of Hb A% is plotted against VCN. The tie bars under the X axes group different specimens from the same individual. Several specimens were harvested and transduced at different times so as to expose the variability in such experiments. (D) Total Hb content before and after treatment with AnkT9W, obtained measuring the areas under hemoglobin peaks detected by HPLC.

As expected, CD34-derived erythroid cells of SCD patients do not synthesize Hb A. A representative example of the Hb synthesis pattern of an untreated SCD sample is shown in Figure 10A (left) and compared to the same sample treated with AnkT9W (right). At baseline 8 SCD samples synthesized on average 26.54%±4.5% of Hb F and 73.46%±4.5% of Hb S, while Hb A was not detected. After treatment with AnkT9W both Hb F and S were significantly reduced (to 19.83%±6.36%, p = 0.001 and to 49.66±13.91, p<0.0001, respectively). On average, Hb A from undetected increased to 30.48%±17.89%, with a mean VCN of 0.98±0.86. The changes in Hb A, F and S% in all 8 samples before and after treatment are shown in Figure 10B from top to bottom, respectively. After treatment with AnkT9W, there was a significant correlation between HbA increase and VCN (p<0.0001, r^2^ = 0.56, Figure 10C), while absolute total Hb content, measured by extrapolation of peak areas, did not change (Figure 10D), indicating that the quality rather than the quantity of Hb determined the phenotypic correction in these cells.

## Discussion

Based on encouraging results in mice [6], [7], [8], [10], [11], [12], [13] and driven by the urgent need for a definitive cure for thalassemia and SCD, a clinical trial was initiated in 2007 [17] using a lentiviral vector that expresses a modified adult β-globin gene (β^A(T87Q)^), flanked by two copies of the 250-base-pair core of the cHS4 chromatin insulator [30] implanted in the U3 region. After gene transfer, the patient became transfusion independent. However, the therapeutic Hb-β^A(T87Q)^ in this patient contributed only one-third of the total Hb synthesized, while Hb E and Hb F accounted equally for the remaining Hb. In addition, about two-thirds of the viral integrations examined contained a deletion of one of the cHS4 cores within the 3′-LTR and the remaining one-third presented a deletion of a cHS4 core within the 5′-LTR. Furthermore, erythroid cells from this patient exhibited a dominant, myeloid-biased cell clone, in which the integrated vector caused transcriptional activation of *HMGA2. HMGA2* upregulation can lead to a clonal growth advantage [31]. The integrated vector introduced a cryptic 3′ splice signal within the cHS4 insulator core and cleavage/polyadenylation within the adjacent R region of the 3′-LTR that triggered alternative splicing of *HGMA2*. This alternative spliced form lacked a distal portion of the *HGMA2* transcript containing multiple binding sites for let-7 microRNAs, which normally controls *HGMA2* degradation [31]. Duplication of the cHS4 core element triggers recombination, deletion of at least one of the two elements and consequent loss of insulating activity that, in concert with the disruption of the full-length *HGMA2* transcript, likely leads to *HGMA2* upregulation. An additional question is whether upregulation of *HMGA2* plays any role in the increased HbF observed in this patient post bone marrow transplant. Therefore, the complications associated with the cHS4 element and upregulation of *HMGA2* suggest that an *in vitro* test, such as the one proposed in our study, could have addressed this question. A thorough analysis of CD34-derived cells, infected with serial dilutions of the vector utilized in this trial, could have indicated the predisposition of the cHS4 to recombine and could have quantified the HbF level prior to bone marrow transplant. Nevertheless it is undeniable that this approach has great potential for success, as evidenced by the fact that this patient no longer needs transfusion therapy, does not manifest any malignant or pre-malignant state and has significantly improved quality of life.

The number and complexity of the mutations within the thalassemic population is a source of great phenotypic variability. This raises concern that some patients, based on their genetic profiles and endogenous hemoglobin production, might be better candidates for transplant than others. This is especially important in light of the intrinsic ability of the specific vectors used in the different clinical trials to make hemoglobin. To date, no study has focused on the correlation between gene transfer and increased hemoglobin levels in patients carrying different β-globin mutations and exhibiting wide phenotypic variability.

To address this important issue we generated a lentiviral vector expressing the human β-globin gene and bearing the ankyrin insulator element. Before using this vector in patient specimens, we tested this vector *in vitro* and in thalassemic mice. In particular, in MEL cells we discovered that gene the ankyrin element is greatly suitable for the purpose of expressing the β-globin. In fact, when associated to the β-globin gene, the ankyrin insulator not only increases its transcription rate but also induces expression at an earlier stage of differentiation in erythroid cells. Furthermore, a higher β-globin mRNA level associated with translationally active ribosomes in cells transduced with AnkT9W, compared to those transduced with T9W, ultimately leads to greater translation of the transgene. In other words, our study suggests that the ability of the various β-globin lentiviral vectors to compensate for the lack of β-globin chain is not only dependent on the relative rate of transcription of the transgenic cassette, but also on its temporal activation during erythroid differentiation. In addition, AnkT9W showed significantly less variability of β-globin synthesis in MEL cell clones containing a single integrated copy, compared to T9W.

As previously reported [18], our results confirm that the ankyrin insulator does not have enhancer-blocking activity. Indeed, we did not observe any reduction in β-globin synthesis in differentiated MEL clones that were transduced with a single copy of T9Ank2W, which carries an ankyrin insulator between the β-globin promoter and the LCR. On the contrary, this vector drives higher synthesis of chimeric Hb compared to T9W, similar to what we measured in cells tranduced with AnkT9W. Therefore, we speculate that an additional ankyrin cassette could be placed between these two elements to maximize β-globin expression, and that an additional insulator/enhancer-blocking element could be introduced into the 3′SIN-LTR. Novel and shorter elements derived from the cHS4 that confer enhancer-blocking activity do not require duplication in order to be effective, and do not decrease the viral titer. Therefore, they could be utilized for this purpose [32]. Furthermore, we observed that this vector was also effective in mice affected by β-thalassemia intermedia when administered via BMT of HSCs pretreated with the lentiviral construct, producing a phenotype more similar to that of WT mice transplanted with normal BM.

Our study was then designed to identify an *in vitro* protocol which could predict correction of both the thalassemic and SCD phenotype in a large pool of patient samples. In this regard, we discovered that AnkT9W increased the synthesis of β-globin protein in most thalassemic and SCD specimens to levels comparable to those observed in carriers and control samples. However, after treatment a subset of thalassemic samples, mostly β0/0, exhibited the lowest amount of total HbA (transgenic and endogenous). In other words, β0/0 specimens required higher amounts of vector to reach therapeutic levels of Hb, whereas β0/+ and β+/+ benefitted from the expression of the endogenous HbA and reached curative levels at lower VCNs. Furthermore, irrespective to the β-globin mutations, additional mechanisms, potentially associated with genetic modifiers and/or other unknown factors, may contribute to the variability observed following gene transfer. For this reason, some β0/0 patients and patients that do not exhibit an increase in HbA proportional to the VCN should be carefully investigated to determine if they would be good candidates for a clinical trial based on this approach. To our knowledge, this is the first study that clearly correlates the genotype, the endogenous levels of hemoglobins and the vector copy number with the phenotypic outcome. Because these findings are clinically relevant, we believe that future clinical trials should take these factors under careful considerations before treating patients. Furthermore, we found that in SCD specimens the treatment with AnkT9W can correct the phenotype by modifying the proportion of sickling versus functional Hb, without changing the overall Hb content.

In general, we believe that analysis of erythroid progenitors transduced with different amounts of lentiviral vectors could be useful for testing the potential of each lentiviral construct prior to BMT.

In summary, our results suggest that clinical trials could benefit from a simple test to predict the relationship between the number of vector copies integrated and the total amount of hemoglobin produced using the erythroid cells of prospective patients. The data presented herein support the use of a simple pre-clinical test to determine the potential efficacy of a given gene therapy treatment in patient-derived cells prior to myeloablation and transplant.

## Materials and Methods

### Human and Animal Ethics

All study participants were enrolled through written consent, according to protocol #05070077971, approved by the Weill Cornell Medical College Institutional Review Board. The periferal blood samples from the SCD patients were obtained during automated red cell exchange as part of their routine clinical care at Montefiore Medical Center. Since the samples were unlinked and deidentified medical waste, the Montefiore Medical Center Institutional Review Board deemed them to be IRB exempt. Experiments conducted on mice were approved by the Weill Cornell Medical College Institutional Animal Care and Use Committee, as described in the protocol #0601-436A.

### Vector production and titration

Viral stocks were generated by co-transfection of the gene transfer plasmid (i.e. T9W, AnkT9W) together with the envelope plasmid (VSV-G), the packaging plasmid (pMDLg/p RRE), and the pRSV-REV vector into 293T cells [6], [33]. An aliquot (5×10^6^) of 293T cells was seeded into cell culture dishes (10 cm) 24 hours prior to transfection in Iscove's modification of Eagle's medium (DMEM, Cellgro, Manassas, VA) with 10% fetal bovine serum, 100 U/ml penicillin, and 100 mg/ml streptomycin, at 37°C under 5% CO_2_. The culture medium was changed 2 hours prior to transfection. The precipitate was formed by adding the plasmids to 450 uL of 0.1× TE (0.1× TE is 10 mM Tris plus 1 mM EDTA) and 50 µL of 2 M CaCl_2_, then adding 500 µL of 2× HEPES-buffered saline (281 mM NaCl, 100 mM HEPES, 1.5 mM Na_2_HPO_4_) dropwise after which the precipitate was vortexed and immediately added to the cultures. The medium (10 ml) was replaced after 16 hours. Viral supernatants were collected at 24 and 48 hours, cleared by low speed centrifugation, and filtered through cellulose acetate (0.2 µm). Following concentration by ultracentrifugation, serial dilutions of concentrated virus (5; 0.5 and 0.05 µL, respectively) were used to infect 1×10^5^ NIH 3T3 cells (ATCC, Manassas, VA) in 1 mL of transfection buffer complemented with polybrene (Millipore, Billerica, MA) at a final concentration of 8 µg/mL. Genomic DNA was extracted after 3 days (Qiagen kit, Valencia, CA). The multiplicity of infection (MOI) was calculated using the following formula: number of cells (1×10^5^)×dilution factor (1 mL/µL viral preparation) X VCN (measured via real-time PCR, using oligos for WPRE element and ID gene, see below).

### MEL cell culture and differentiation

Murine erythroleukemia cells [34] were grown in DMEM (Cellgro), supplemented with 10% FBS (Hyclone, South Logan, UT) and 1% penicillin/streptomycin. Erythroid differentiation was achieved by seeding 2×10^6^ cells, in log phase, into 3 mL of fresh culture media containing HMBA, at a final concentration of 1 mg/mL. Treatment with HMBA was repeated after 2 and 4 days of culture, cells being collected for analysis after 6 days and every day for time-course analysis.

### Southern blot analysis

For identification of viral genomic stability genomic DNA from MEL cells was isolated, digested with *Xmn*I, and investigated by Southern blot analysis using a [^32^P]dCTP-labeled *Nco*I/*Bam*HI probe from the human β-globin gene. Since this fragment shares more than 90% homology with the mouse β-globin gene, both the human (top) and mouse (bottom) β-globin gene hybridized to this probe.

### Generation of bone marrow transplanted chimeras

Murine bone marrow cells were harvested from WT and Hbb*^th3^* ^/+^ donor mice (Jackson Laboratories, Bar Harbor, ME). Bone marrow cells were resuspended in X-VIVO-15 serum-free medium and supplemented with 10 ng/mL IL-1α, 100 U/mL IL-3, 150 U/mL IL-6, 10 ng/mL Kit ligand obtained from Genzyme (Cambridge, MA), 0.5 mM β-mercaptoethanol obtained from Sigma (St Louis, MO), 200 mM L-glutamine, 100 IU/mL penicillin, and 100 µg/mL streptomycin. Cells were then pelleted and resuspended in serum-free medium containing concentrated lentiviral supernatant, supplemented with 4 mg/mL polybrene obtained from Sigma, 200 mM L-glutamine, 100 U/mL penicillin, 100 µg/mL streptomycin, and cytokines as above, and cultured for 8 hours. Transduced and control cells (2×10^6^) were then intravenously injected into each of the irradiated female recipients to establish bone marrow chimeras. Recipient mice (8- to 10-week-old C57/BL6 mice) were irradiated with 12 Gy (split dose 2×6 Gy) on the day of transplantation.

### PCR and Real Time (RT)-PCR

Insertion of the ankyrin insulator at both viral LTRs was confirmed by PCR using two sets of oligonucleotides annealing within the ankyrin insulator and a region of the viral genome present exclusively at the 5′ or the 3′ sites. To amplify the ankyrin in the 5′ LTR we utilized the following oligonucleotides: ankyrin/LTR5′-FW 5′-TGCCCCGGATGTAGGCATGCG-3′, and LTR5′-REV 5′-CGCCATGCTAGAGATTTTCCACA-3′, which anneals within the viral sequence in the primer binding site. To amplify the ankyrin in the 3′ LTR we utilized the following oligonucleotides: LTR3′-FW 5′-ACTGGAAGGGCTAATTCACTCCCA-3′ which anneals within the NEF viral sequence, and Ankyirin/LTR3′-REV 5′-AGGTGCTCTTGTAATCTGCGGT.

Retrotranscription of total mRNA was done using the SuperScript™ II First Strand Kit (Invitrogen, Carlsbad, CA). Q-PCR reactions were performed using the ABI Prism 7700 Sequence Detection System (Applied Biosystems, Foster City, CA), with either TaqMan (TaqMan PCR 2× Master mix; Applied Biosystems) or SYBR Green (iTaqTM SYBR® Green Supermix, Bio-Rad Laboratories, Hercules, CA) chemistry. Quantitative real-time PCR assays of globin transcripts were carried out using gene-specific double fluorescently labeled probes. The following primer and probe sequences were used (forward, reverse and probe of each gene, respectively): β: Fw: 5′-CAAGAAAGTGCTCGGTGCCT-3′; Rev: 5′- GCAAAGGTGCCCTTGAGGT-3′; 5′-FAM-TAGTGATGGCCTGGCTCACCTGGAC-TAMRA-3′; α: Fw: 5′-TCCCCACCACCAAGACCTAC-3′; Rev: 5′-CCTTAACCTGGGCAGAGCC-3′; 5′-FAM-TCCCGCACTTCGACCTGAGCCA-TAMRA-3′. For real-time PCR of the reference genes, we used as an endogenous control the human glyceraldehyde-3-phosphate dehydrogenase (GAPDH) kit, in which the probe is fluorescently labeled with VIC (Applied Biosystems). The number of integrations (VCN) was quantified by Q-PCR using oligos (Fw: 5′-CGGCTGTTGGGCACTGA-3′; Rev: 5′-GGAAGGTCCGCTGGATTGA-3′) and a probe (5′-FAM-ATGGCTGCTCGCCTGTGTTGCC-TAMRA-3′) for a specific sequence present in the vector (WPRE) and compared it to an endogenous control present in two copies within the genome (ID-1 Fw: 5′-AAGGTGAGCAAGGTGGAGATTC-3′; Rev: 5′-TTCCGAGTTCAGCTCCAACTG-3′).

### Two-phase liquid cultures, benzidine staining and transduction

Consented patients with β-thalassemia major, β-thalassemia carriers and healthy individuals donated between 20 and 30 mL of peripheral blood. To isolate ErPCs or CD34^+^ cells, we first separated the mononuclear fraction by Ficoll-Hypaque density gradient. We selectively expanded ErPCs by seeding them into α-modified essential medium (α-MEM) containing 10% fetal calf serum (Biological Industries, Beit-Haemek, Israel), streptomycin (100 U/mL), glutamine (100 mg/mL), stem cell factor (10 ng/mL) and (1 µg/mL) cyclosporine A (Sigma, St. Louis, MO). Alternatively, we selected CD34^+^ cells by immunomagnetic separation, using the CD34 microbeads kit (Miltenyi Biotec Inc., Auburn, CA) and then expanded these cells following a modified version of the protocol described by Leberbauer and colleagues. Cells were seeded in 5 mL of serum-free StemSpan with 50 µL of StemSpan CC-100 cytokine cocktail (both from Stemcell Technologies, Vancouver, BA, Canada), 2 U/mL Erytrhopoietin (Amgen, Thousand Oaks, CA), 10^−6^ M dexamethasone (Sigma) and 1% penicillin streptomycin. CD34^+^ cultures were kept undifferentiated by refreshing the medium twice a week and density gradient centrifugation was used to remove both dead and spontaneously differentiating cells. At day 10, cells were analyzed by flow cytometry showing a homogenous population characterized as being C-kit^+^, CD34^+^, CD235a^−^ and Band3^−^ (not shown). At this stage cells were either frozen (with 50% chracterized Hyclone FBS, 10%DMSO, Sigma, and 40% Iscove's Modified DMEM, Cellgro), or used for experiments. After 5 to 7 days (for the ErPCs) or 3 weeks (for the CD34^+^ cells) in phase I, cells were transferred into phase II media containing α-modified essential medium supplemented with 30% fetal calf serum and 10^−5^ M β-mercaptoethanol. Erythropoietin was added (10 U/mL) to stimulate erythroid differentiation. Cells were infected with serial dilutions of the viruses, starting with MOI 0.3 and multiples of it. Cells were collected on day 10 of phase II for all analyses. The level of differentiation was assessed by benzidine staining. Briefly, 1 mL of a solution made by adding 1 g of benzidine dihydrochloride to 14.6 mL of glacial acetic acid was mixed with 20 mL of 33% H_2_O_2_. Cells were mixed (at a 1∶1 ratio) with the activated benzidine and immediately scored for blue staining in a hemotocytometer.

### High performance liquid chromatography (HPLC)

Cell pellets were lysed with HPLC-grade water and loaded into a System Gold 126 Solvent Module instrument (Beckman Coulter, Fullerton, CA). Hbs were separated on a cation-exchange Synchropak CM300 column (Eichrom Technologies, Inc, Darien, IL), and detected at a wavelength of 415 nm. The Hbs were bound to the column with mobile phase B (10 mmol/L Bis-Tris, 1 mmol/L KCN, pH 6.3) and eluted with mobile phase A (10 mmoI/L Bis-Tris, 1 mmol/L KCN containing sodium acetate trihydrate, pH 6.15). Serial dilutions of a solution with known concentrations of Hb A and Hb F (Analytical Control System, Inc, Fishers, IN) were used to generate a calibration curve, where the absorbance detected at 415 nm was plotted against the concentration values. Types and quantity of Hbs in samples were assessed by comparison to standard hemoglobin controls.

### Polysomal analysis

Polysome fractions were isolated on sucrose gradients. The amount of mRNA in each fraction was determined to evaluate the association between the ribosomes and endogenous or transgenic β-globin mRNAs. Cells were homogenized in lysis buffer (100 mM NaCl, 10 mM MgCl_2_, 30 mM Tris-HCl, pH 7.5, 1 mM DTT, 30 U/ml RNasin) supplemented with 1% Triton X-100. After incubation on ice for 5 minutes, the lysates were centrifuged at 4°C for 10 minutes at 12,000×*g*. The supernatants were then loaded on a 10%–50% (w/v) sucrose gradient and sedimented by centrifugation at 34,000 rpm for 180 min using a Beckman SW41 rotor. Each gradient was separated into 13 fractions. RNA was extracted from each fraction and analyzed by RT-PCR.

### Statistical analyses

For two-group comparisons we used either paired or unpaired t-tests (where normal distributions and equal variances were met) or Mann Whitney and Wilcoxon tests. For comparison of three or more groups, we computed means with a one-way Anova test (for samples with normal distributions and equal variances) or medians with a non-parametric Kruskal-Wallis test. Correlation between variables was evaluated by linear regression. All tests were done using GraphPad Prism software, version 5.0a.
